# RESPONSE TO THE DECADE OF HEALTHY AGING: AN EXEMPLAR ICOPE-BASED CARE MODEL

**DOI:** 10.1093/geroni/igae098.0542

**Published:** 2024-12-31

**Authors:** Doris Sau Fung Yu, Yiting Tang

**Affiliations:** Chair; Discussant

## Abstract

The United Nations Decade of Healthy Aging 2021-2030 calls for proactive and collaborative strategies to enable older adults to “live well” with optimized functional capacity, considering both intrinsic capacity (IC) and the environment. The Integrated Care of Older People (ICOPE) framework has been established as a comprehensive approach to guide IC assessment and care prescription for promoting healthy aging. In line with this global initiative, Hong Kong, a city with accelerated aging, has implemented the “Jockey Club Pathway to Healthy Aging” (Path-HA) program. This 12-week program incorporates the concepts of clinical critical care pathway, goal-oriented empowerment-based behavioral modification, asset-based capacity building, and health-oriented peer support. The program aims to optimize the functional status of local older adults across Hong Kong districts. The signs of accelerated aging and risk factors related to ICOPE health concerns are identified through the assessments. To date, more than 5,500 older adults are involved in this study and more than 1,800 have been recruited into the program and have received person-centered care and interventions for reducing the risks corresponding to critical pathways to healthy aging. This symposium is aligned with the ICOPE framework to address healthy aging in Hong Kong: (a) IC profiles of older adults, (b) promoting IC with a synergistic way to enhance care prescription, (c) critical pathways of the Path-HA program, and (d) the effects of critical care pathways on IC conditions. With these efforts endeavored, we will share the outcomes, insights and foresight regarding the regional contribution to optimizing functional capacity.

